# Experience and practice of the Emergency Operations Center, Chinese Center for Disease Control and Prevention: a case study of response to the H7N9 outbreak

**DOI:** 10.1186/s40249-020-00789-x

**Published:** 2021-01-06

**Authors:** Fan Ding, Qun Li, Lian-Mei Jin

**Affiliations:** Public Health Emergency Center, Chinese Center for Disease Control and Prevention, Beijing, China

**Keywords:** Emergency Operations Center, Practice, Response, Plan, Human avian influenza A (H7N9)

## Abstract

**Background:**

Emergency Operations Center (EOC) is a place to provide response to public health emergencies. Chinese Center for Disease Control and Prevention (China CDC)’s EOC was officially established in 2016, which has been the core department for the public health emergencies and risk response. In recent years, we have been continuously improving the function of EOC through many incidents. In the study, we hope to share the construction status, operation management experience of China CDC’s EOC and the response process in the human avian influenza A (H7N9) outbreak.

**Main text:**

The China CDC’s EOC mainly focus on building the five core elements including sites/places and facilities, information and data, plans and procedures, training and exercises, and logistics. Based on summarizing previous emergency response, the China CDC’s EOC established its own incident management and the standardized response procedures. The event-specific data, context-specific data and event management data could be obtained through various source. The logistics department of the EOC also provides comprehensive support. The well-trained staff is another necessary conditions for its operation. Through sharing the response process of H7N9 outbreak, it further explains the EOC’s functions in the five phases of outbreak response, such as the formulation of the incident response framework, monitoring, personnel dispatch and resource mobilization.

**Conclusions:**

The EOC contributes to faster and more efficient responses during emergencies which enable a greater reduction in morbidity and mortality. Compared with the traditional incident response process, under the command and coordination of China CDC’s EOC, each group involved in the response has a clearer goal, responsibilities and tasks at each stage. Meanwhile, each group also gave full play to its own expertise and advantages. As a whole, incident response tended to be more specialized and precise, which generally improves the efficiency of incident response. However, different countries and regions have different response processes to the events. We still suggested that appropriate emergency operation plan should be made according to the complexity of incident response in the region when constructing response mechanism, through our experience. And the China CDC’s EOC is still at growing and groping phase.

**Graphic abstract:**

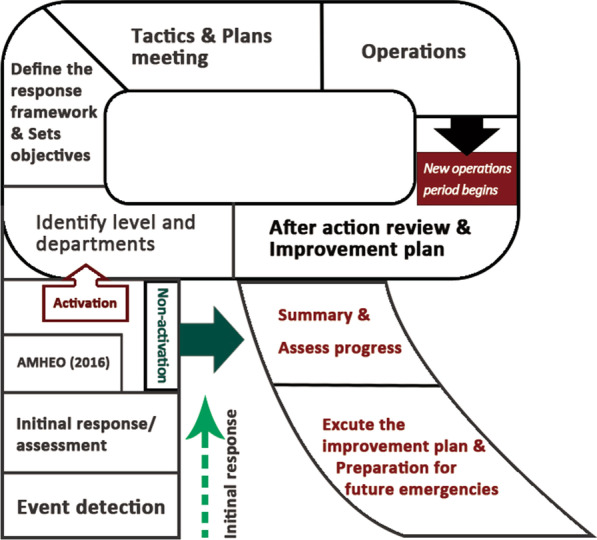

## Background

For Emergency Operations Center (EOC), different organizations/agencies have different definitions. However, Federal Emergency Management Agency (FEMA), Centers for Disease Control and Prevention of the USA (USCDC) and World Health Organization (WHO) all emphasize that EOC is a place to provide emergency response for professionals dealing with public health emergencies [1]. It can command and coordinate relevant information and resources, and manage emergencies. We all believe that an effective EOC should be an organic combination of infrastructure and functionality. In China, The EOC is a place where Centers for Disease Control and Prevention (CDCs) organize and conduct the related emergency response work. It is the place for emergency preparedness, daily operation and emergency response. It can be a dedicated place, or a space that is used in conjunction with other agency functions, or it can be a virtual place realized through information-based facilities.

The EOC of China CDC was officially established in 2016, as a branch of the Public Health Emergency Center (PHEC) of China CDC, which has become the core department for the public health emergencies and risk response. According to Administrative Measures for Health Emergency Operations (AMHEO) [2], China CDC’s EOC fully plays a role in command and coordination, resource integration, risk communication and technical support in event response.

A functional public health EOC is an important component of meeting the International Health Regulations (IHR) [3] requirements. In 2012, WHO established the Public Health Emergency Operations Centre Network (EOC-NET) in order to facilitate the establishment of global PHEOC [1, 4]. From 2015 to 2016, the National Health Emergency Working Standards for Centers of Disease Control and Prevention (trial) [5] and the 13th Five-year Plan for the Prevention and Treatment of Infectious Diseases [6] issued by the National Health Commission (NHC) of the People’s Republic of China (PRC) clearly put forward the requirements for the construction of EOC in China.

So that EOC became an emerging field of practice in China. It is necessary to effectively manage complex public health risk or threats by utilizing specific knowledge, technology and organizational principles found in incident management [2, 3]. During recent years, the CDCs at all-levels across the country have been exploring the model of health emergency operation management, and the construction of EOC has been carried out to achieve the health emergency interconnection, resource integration and information sharing.

### EOC’s role in different situations

EOC is usually been used by jurisdictions and organizations as one of the most important elements in their emergency management programs. It is the location where staff from multiple agencies typically come together to address imminent threats and hazards and to provide coordinated support to incident command, on-scene personnel, and/or other EOCs [1, 4]. A growing number of countries agree that, it should be increasingly viewed as the necessary component of emergency preparedness and used for multiagency coordination and response to a variety of hazards, including natural disasters, chemical spills, radio nuclear incidents, humanitarian emergencies, and disease outbreaks [2, 3, 5, 6].

The operations of EOC is based on the concept of a comprehensive and systematic incident management method, including event command and coordination, resource management and information management [3]. Within the necessary framework, five basic functions are usually established: management, operations, planning, logistics, finance and administration, while maintaining flexibility to adapt to different events, institutions and jurisdictions [4]. The adoption of a common organizational model or framework for emergency management at all levels, from the country to the primary health services, is of great benefit. According that, the main functions of China CDC’s EOC showed in Box 1.

Box 1: Main function of Emergency Operations Center (EOC) of China CDC

**Table:** 

**During routine work**, the emergency preparedness duty should be carried out to continuously event surveillance and risk assessment **When event outbreaks**, the EOC would be activated depending on the scale of the events. And the function of EOC contains recourse coordination, surveillance strengthening, coordinating resources, providing epidemiological technical services, etc **After the response completed**, the EOC should conduct the evaluation of the event response process, summarize the problems in the event response process, and propose improvement plans. The purpose of improving emergency operation capability is to correct problems in the future through training and exercise

This paper summarizes the construction experience of China CDC’s EOC, as well as the case statement of coping with threats. And it is hoped to better understand the public health response in China.

## Core elements of China CDC’s EOC

In China CDC, we focused on building the following five core elements of the EOC, including sites/places and facilities, information and data, plans and procedures, training and exercises, and logistics.

### Physical site and facility integrating management and function

According to the 13th Five-year Plan for the Prevention of Infectious Diseases (2016–2020), China has clearly put forward the practice for the construction of EOCs. In the years leading up to 2010, China CDC initially established the EOC which contains a main hall and some function area for small meetings and discussions, and introduced the remote conference system to realize the remote consultation [7, 8]. In addition, the teleconference system and light emitting diode (LED) information displaying system are also introduced to further the information display environment. However, the EOC is more than just a physical place. A conference room or a command center is not an EOC, and the dispatch of emergency personnel is not an EOC either [2, 3]. The EOC should be an organic combination of sites and emergency management functions, which can be a fixed place, a temporary facility, or a virtual architecture. Employees can participate remotely. The EOC should be positioned as a focal point for coordinating resources, information and communications for data reception, integration, analysis and interpretation, and coordination, with less focus on physical infrastructure. Therefore, the lack of a dedicated physical location has not hindered EOC's operations. The construction of EOC should be demand-oriented.

### Rapid and efficient response plans and procedures

Based on severe acute respiratory syndrome (SARS), human avian influenza A (H7N9), and other infectious diseases outbreaks, as well as various natural disasters and man-made disasters, China CDC has built its own model of incident detection, reporting and response processes. According to different events, we developed different technical, hazard-specific response and support plans, manuals and handbooks [9, 10]. In order to maintain consistency with WHO and other countries, China CDC compiled and issued a comprehensive plan at the end of 2016, called AMHEO, which is the combination of the emergency operation plan (EOP) and EOC plan. It contains the incident management and the standardized response procedures. The EOC can be activated in response to all kind of public health emergencies including the natural or man-made disasters, infectious disease outbreaks, and other emergencies. Besides watching level and alert level, there are three different levels of activation (see Table 1), depending on the scale of the event. In addition, the China CDC is also developing a series of incident action plan (IAP) and the standardized forms for each incident.

**Table 1:** Health emergency practice launched by China CDC’s EOC during 2017–2018

Period	Outbreak/disaster	Type	Location	Response level^a^	Comments/action taken
January–July, 2017	H7N9	Infectious disease outbreak	22 provinces, China	III	Coordinated departments to develop the epidemiological and lab test strategy
August–October, 2017	Earthquake	Nature disaster	Sichuan and Xinjiang, China	II	Deployed team to the field for the public health prevention and control
November, 2017– January, 2018	Plague	Infectious disease outbreak	Madagascar	II	Deployed team to the field for the public health prevention and control
July–Sepetember, 2018	Flood	Nature disaster	Sichuan and Gansu, China	III	Deployed team to the field for the public health prevention and control
July–October, 2018	Group events caused by substandard vaccines	Infectious disease outbreak	Shandong and Jilin, China	I	Assess the risk of problematic vaccine and develop reseeding strategies
July–October, 2018	Polio virus	Infectious disease outbreak	Xinjiang, China	III	Strengthen sampling and case monitoring, formulate vaccination strategies

^a^There are three different levels of China CDC’s response depending on the scale of the event. (i) Level III is the lowest level of response. Only one or two subject matter department lead to the response with their staff. However, EOC would not be activated. (ii) Level II involves more than two departments staff, or the relevant area and resources from the China CDC. Time-sensitive tasks and needs may extend beyond core business hours. EOC staff may lead or assist with the response. (iii) Level I is the highest level, requiring all agency-wide effort

**Fig. 1:**
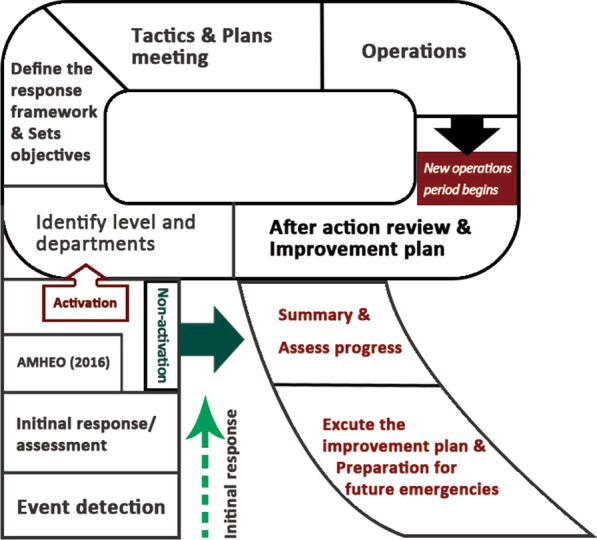
Operational period cycle from the event detection to the end of response and the preparation for the future emergency. *AMHEO* Administrative Measures for Health Emergency Operations

When an incident is detected, an initial verification and assessment would conduct to determine whether an emergency response needs to be initiated. If necessary, the emergency response process would be activated according to the AMHEO which includes: (i) determination on the response level and department, (ii) establishment of the emergency operation structure, (iii) setting the response goals and tasks, (iv) clarification of the response strategy and plan, and (v) then implementing the emergency operations. Some events may go into the second round of the workflow circle. At some stage of the event response process (usually in the middle of the response process) or at the end of the process, the after action review (AAR) should be organized and the improvement plan drafted. After the event response, the summary and assessment on the response process should be carried out, and the contents need improving should be listed according to improvement plan, as well as the preparation for future event response should be made (Fig. 1).

EOC should also function during non-outbreak periods, and surveillance data should routinely be interpreted by an epidemiologic workforce. Like USCDC’s EOC, the slogan is 7 days, 24 h [11–13]. Such an “always on” EOC facilitates the rapid transition to response model during outbreaks and improves the cost-effectiveness of the infrastructure investment. But this is the most fully functional EOC and China CDC is on the way to this direction. Routine use of EOCs during outbreaks and non-outbreak periods helps ensure sustained technical capacity for data analysis and interpretation, and usage of visualization tools and equipment [2–4]. This EOC model would meet the global public health response need.

### Access and utilization of information

Information is the lifeblood of an EOC [2, 3, 5–7, 14–18]. According to the activities and tasks, EOC has specific information needs and require various sets of data. EOC also need to collect and analysis data and information for operation. Three types of data, including event-specific data, context-specific data and event management data, are usually obtained. Event-specific data can reflect information on what, how, where, who, and the current status of events. The sources of information may come from public health emergency management system, which focused on information of the emergencies and health risk factors, and the field investigation recourses. Context-specific data can reflect background information such as geographical information, demographic information, environmental information, health resources information, emergency shelter, the incidence of related diseases and health services. Event management data are organized for the functional domains (management, operations, planning, logistics, finance and administration) in the EOC. Such data may include humanity resources, equipment, the status of interventions, partner activities, resource deployments, expenditure progress on achievement of objectives, and so on. Besides, we also keep abreast of the current situation of staff information, expert database and logistics supplies.

In China CDC, the branch for logistics takes the responsibility to the information supply and update for EOC. With the support of the logistic department, EOC can coordinate the relevant personnel and other resources timely and efficiently in the emergencies. Moreover, the Emergency Operations Management Information System (EOMIS) of China CDC is about to be online. In the nearly future, it will be more conveniently and efficiently to manage the emergency resources.

Collecting, processing and sharing information in time is the key function of EOC [12–14]. Information can be presented in a variety of ways. Information should be collected according to local conditions. In particular, EOCs monitor epidemiologic data and field reports from a variety of sources using data technologies and informal networks of public health professionals during activation [2, 4]. Ideally, information collection should be based on standardized terminology and general data structure, and should be able to exchange horizontally and vertically. However, it may not necessarily to have an emergency operations management system for information management. The content and process of information system construction should comply with the requirements of information management. In practice, which kind of methods is more suitable for information management, still needs to be further assessed.

### Well trained professionals

Well-trained staff is one of the necessary conditions for EOC’s operation. Training should be continuously strengthened for staff in future. EOC can coordinate a variety of the information and resources. When the EOC activated, it should be staffed with a team of subject matter experts, analysts, logistics staff, and support staff [19]. The function and staffing of the EOC should be assessed through an ongoing series of training and exercises [2–5]. Well-trained professionals are the key to the operations of EOC. Trained experts who know what to do are critical to building a functioning EOC [7–10]. Training should be purposefully conducted according to the capabilities required by the EOC. In China CDC’s EOC, how to use the incident management system, special post training for working group of EOC, and professional technology, skills and practical training are the most three important training content. In addition, all personnel should have the information and communication technology (ICT) skills required to work in the EOC. The training targets of EOC mainly include all kinds of personnel who may participate in emergency response. The training can be carried out according to different professions, positions and levels. Training methods can be used in a variety of styles including face-to-face or online teaching, internships, etc. The training frequency is determined by the needs. Before and after the training, the trainees should be assessed to confirm whether the training objectives are achieved and whether their abilities meet the needs of participating in emergency operations. According to the evaluation results, the training program can also be improved.

Exercise is a primary training tool. The abilities of professionals should be consolidated and improved. The two types of exercises—discussion-based and operations-based exercises are usually used in the daily activity in the EOC [11, 19–21]. In practice, the various exercise types may be modified or combined in order to meet specific objectives, especially when resources are limited.

### Initiatives in public health emergency management

Through the adjustment of health emergency practice and EOP in 2016, China CDC’s EOC established the new response procedure. The health emergency practice launched by China CDC’s EOC during 2017–2018 shown in Table 1.

## Human avian influenza A (H7N9) epidemic during January–July 2017

**Fig. 2:**
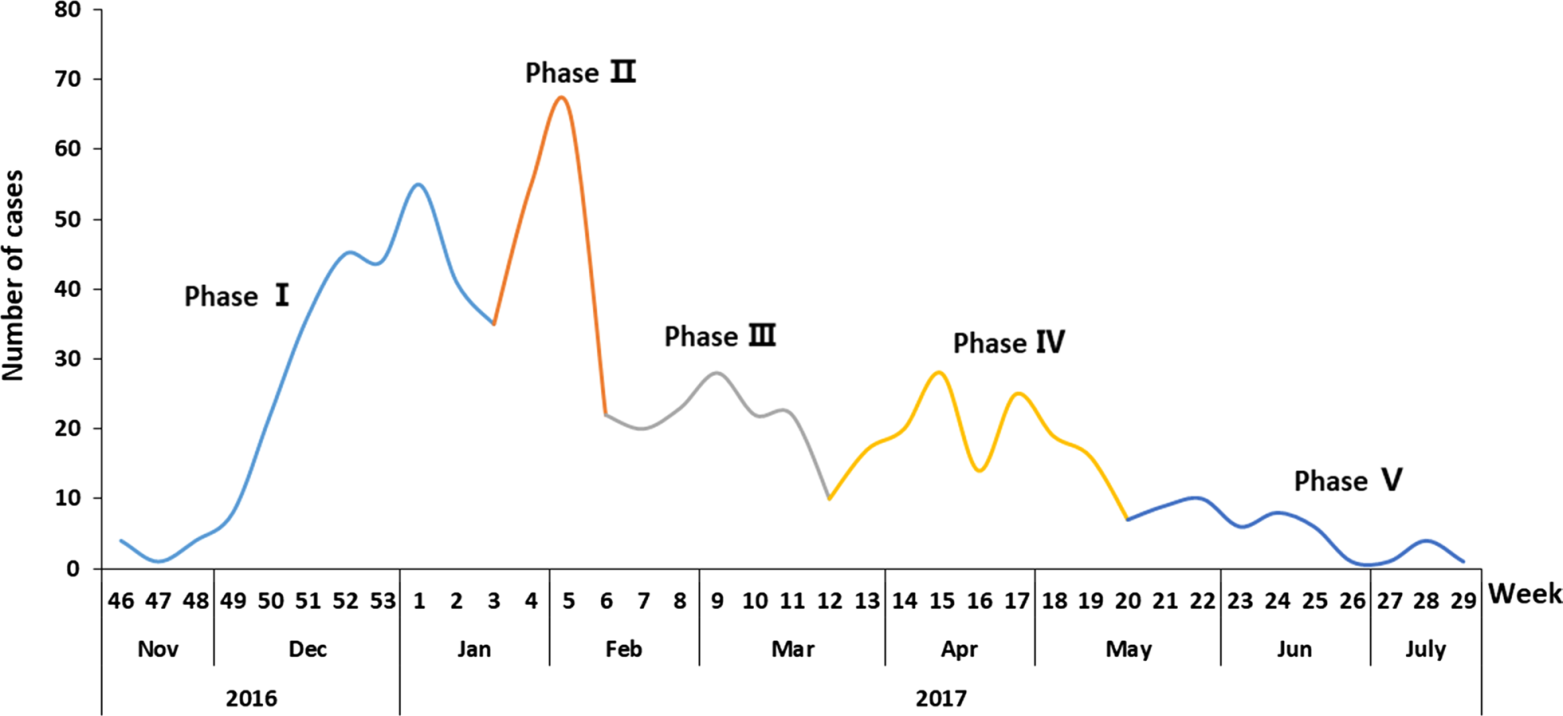
Time distribution of H7N9 cases and the different response stages

The epidemic has taken nearly half a year. Since December 2016, the reported H7N9 case number has been significantly higher than the same period in the past in China. As of January 8, 2017, 169 confirmed cases and 44 deaths have been reported [12]. The epidemic has spread to 10 provinces. The confirmed cases have increased by 6.34 times compared with the same period in last two years, as well as the number of deaths has increased by 5.29 times. The response process of this outbreak can be roughly divided into the following five stages (Fig. 2).

### Phase I: From the 46th week of 2016 to the 2nd week of 2017

Through the China CDC’s infectious disease information reporting system, it shows the number of human cases of H7N9 has been rising rapidly in several provinces. During this period, the number of cases per week increased from 4 to the top of 55. And the nationwide total number of the reported cases was up to 260 from November 7, 2016 to January 15, 2017. Given the situation, it was expected that China CDC will continue to carry out intensive surveillance, connected with the field and further verify the situation, and technical support services for provincial human infection with H7N9 avian flu. After risk assessment, China CDC’s EOC has been activated with a level III response for the H7N9 influenza outbreak. Subsequently, EOC organized the meetings on strategies and technical issues related to the epidemic response. And then the plan, objectives and periodic tasks of this response were determined. Functional groups have been set up for monitoring, control strategy, field management, risk communication and logistics. Incident action plan (IAP) was determined according to the responsibilities of each group.

### Phase II: From the 3rd week to the 5th week of 2017

The number of cases per week in January 2017 has risen sharply to 66 across the country, mainly in provinces along the Yangtze River, with 16 provinces reporting H7N9 cases. In the process of response, EOC gave full play to the role of resource integration and coordination, and mobilized professionals of the relevant provincial CDC to jointly handle the epidemic. The field teams have been sent to several provinces to investigate the reasons for the sharp increase in the number of reporting cases. And the comprehensive coordination group organized daily consultation meetings to provide epidemiological technical support to the field teams. The Chinese traditional festival, the Spring Festival, was in this stage. It is speculated that the increase of the case number might be related to live poultry consumption increase in the early part of the festival. And the rapid decline trend in a short period after the holiday is not only because the live poultry market management measures adopted in various places, but also might be closely related to the natural closure of the market during the Spring Festival.

### Phase III: From the 6th week to the 11th week of 2017

The number of cases in the previously high-incidence provinces along the Yangtze River continued to decline from February to March, 2017. The number of cases fluctuated around 20 per week. The epidemic has moved from the coastal provinces of southeast China to south and southwest China. Through remote video consultation, the epidemiology group regularly carries out discussions and studies on the epidemic situation in 16 key provinces. Suggestions on prevention and control measures should be given according to the particularity of different regions. In addition, EOC sent infectious disease teams to the newly affected provinces for investigation, and the logistics team provided on-site supplies and equipment. The risk communication team compiled risk communication materials for important epidemic data and prevention and control information, and timely release them to the National Health Commission, the media and the public. According to the work task arrangement of this stage, the comprehensive team organized regular meetings in time to adjust the emergency operation plan, so as to ensure the efficient completion of the prevention and control objectives of this phrase.

### Phase IV: From the 12th week to the 19th week of 2017

A total of 22 provinces reported cases during this period, but the number of cases continued to remain at a low level from March to May 2017. The per week number of cases continued to fluctuate around 20. The epidemic is spreading to north, northwest and southwest China. In addition to strengthening the surveillance of the epidemic situation across the country, the epidemiological team of the China CDC’s EOC had also conducted regular communication with the whole country and some key provinces, and actively shared the experience and strategies for the prevention and control of H7N9 nationwide. The risk communication team also organized experts to further develop measures to protect the public. The comprehensive group once again took the lead in organizing and adjusting the prevention and control response targets and priorities at this stage.

### Phase V: From the 20th week to the 29th week of 2017

During this period, a total of 17 provinces have reported cases, with no more than 5 cases reported in each province. The epidemic level has been further reduced compared with the previous period, and has basically returned to normal. However, the number of cases is still widespread. The China CDC recommended that local authorities continue to maintain controls on live poultry markets and monitor cases. EOC organizes each group to study and evaluate the epidemic trend and risk assessment at this stage, and suggested that the level-III response for this epidemic should be terminated. Besides, PHEC will continue to carry out continuous surveillance of the H7N9 epidemic. The comprehensive coordination team worked with emergency operations supervisor would take the hotwash and AAR for this response process and form the improvement plan**.**

During the process, the Chinese Field Epidemiology Program (CFETP) fellow served as the liaison between the EOC and field provinces. The PHEC coordinated seamless communication between the CDCs’ laboratories. When the EOC was deactivated in June 2017, none of the human contacts had tested positive. Through our 7-month around epidemiological action, the epidemic had been controlled.

## Conclusions

Through countless emergency response examples, it is shown that increasing the international community’s ability to rapidly and effectively respond to public health threats ensures the broader global health security of all people. In resource-limited situation, emergency response is centered on achieving the biggest public health impact. In China CDC, the EOC is no longer just a centralized office, but a comprehensive functional center that integrates various resources, information and mechanisms. And it contributes to faster and more efficient responses during emergencies which enable a greater reduction in morbidity and mortality. According to the H7N9 epidemic response process, it could be confirmed that the EOC can better organize and allocate various resources so as to achieve the purpose of standardized handling. Compared with the traditional incident response process, under the command and coordination of China CDC’s EOC, each group involved in the response has a clearer goal, responsibilities and tasks at each stage. Meanwhile, each group also gave full play to its own expertise and advantages. As a whole, incident response tended to be more specialized and precise, which generally improves the efficiency of incident response. However, different countries and regions have different response processes to the events. It is suggested that appropriate EOP should be made according to the complexity of incident response in the region when constructing response mechanism.

Besides, it is all the more necessary to strengthen close collaboration and partnership with international organizations to enable more to be accomplished through leveraging individual institutional strengths. The standardize approach to respond to public health emergencies meet global standard’s needs. With these efforts, we aims to reach the goal of saving lives and protecting people while making the world a safer place from disease outbreaks and other public health threats.

Beyond that, with the Belt and Road Initiative, China's public health services should also go abroad to help other international partner in need. We need to improve the operational mechanism and the function of EOC. Efforts to strengthen EOC capacity must build on existing emergency response structures. Any augmentation of technology and infrastructure also should improve non-emergency capability to be sustainable and effective.

## Data Availability

All data supporting the findings of this study are included in the article.

## Abbreviations

EOC: Emergency Operations Center
FEMA: Federal Emergency Management Agency
USCDC: Centers for disease Control and Prevention of the USA
WHO: World Health Organization
PHEC: Public Health Emergency Center
AMHEO: Administrative Measures for Health Emergency Operations
NHCPRC: National Health Commission of the People’s Republic of China
IHR: International Health Regulations
LED: Light emitting diode
SARS: Severe acute respiratory syndrome
H7N9: Human avian influenza A
EOP: Emergency operation plan
IAP: Incident action plan
IP: Improvement plan
AAR: After action review
EOMIS: Emergency Operations Management Information System
ICT: Information and Communication Technology
